# Selective Curettage and Two‐Segment Restoration in the Management of a Palatogingival Groove With Vital Pulp—A 2‐Year Follow‐Up Case Report

**DOI:** 10.1155/crid/9391532

**Published:** 2026-08-27

**Authors:** Shuting Zeng, Simin Guo, Zilong Deng, Rongyang Ma, Wanghong Zhao

**Affiliations:** ^1^ Department of Stomatology, Nanfang Hospital, Southern Medical University, Guangzhou, China, fimmu.com; ^2^ Department of Stomatology, Shenzhen Second People′s Hospital, Shenzhen, Guangdong, China, szrch.com

**Keywords:** microsurgery, palatogingival groove, through-and-through lesion, tooth developmental anomaly, vital pulp preservation

## Abstract

This case presents a novel microsurgical approach for managing a severe palatogingival groove (PGG) while preserving pulp vitality. A 29‐year‐old male exhibited a 10‐mm palatal pocket, buccal sinus tract, and an extensive through‐and‐through cortical plate defect on the maxillary right lateral incisor. Despite the PGG extending 6 mm apically, pulp sensibility remained normal. A conservative microsurgery featuring “selective curettage” was performed to preserve the apical neurovascular bundle. The defect was sealed using a two‐segment restoration: iRoot BP Plus radicularly and composite resin coronally. At the 2‐year follow‐up, the patient was asymptomatic with normal probing depths (< 3 mm), complete sinus tract resolution, substantial alveolar bone healing, and maintained pulp vitality. Combining selective curettage with a two‐segment restoration offers a predictable, biologically driven strategy for simultaneous periodontal regeneration and vital pulp preservation.

## 1. Introduction

The palatogingival groove (PGG) is a developmental malformation that typically affects the palatal root surfaces of maxillary incisors, with an incidence of 2.8%–18% [1–3]. Typically originating at the cingulum, these grooves extend apically across the cementoenamel junction to terminate at variable root depths [4, 5]. Its clinical significance depends on its morphology (depth, length, and complexity), which dictates the risk of localized periodontitis [6] and combined endodontic‐periodontic lesions via accessory canals [7]. Although various classification systems exist for PGGs, the most recently developed Gu′s classification, based on micro‐CT, categorizes them into three types [8]: Type I (superficial, confined to the coronal third), Type II (extending deeper with a simple canal), and Type III (deep grooves with complex canals).

Lacking consolidated guidelines, PGG management is multidisciplinary and dictated by pulpal status and groove complexity [9]. For vital teeth, treatments aim to eliminate the defect, ranging from conservative curettage and sealing to advanced periodontal surgery or guided tissue regeneration (GTR). Conversely, necrotic pulps require combined endodontic‐periodontal therapy, with extraction reserved for severe cases [10, 11].

This report presents a case of PGG with a through‐and‐through cortical plate defect, in which pulp vitality was preserved using a microsurgical approach involving selective curettage and a two‐segment restoration.

## 2. Case Report

A 29‐year‐old Chinese male patient was referred to the Department of Endodontics with a chief complaint of gingival swelling and a persistent pustule on the labial aspect of the right anterior maxilla for 2 weeks. The patient presented with a deep overbite and prior orthodontic treatment. His medical, trauma, familial, and psychosocial histories were entirely noncontributory.

**Figure 1:**
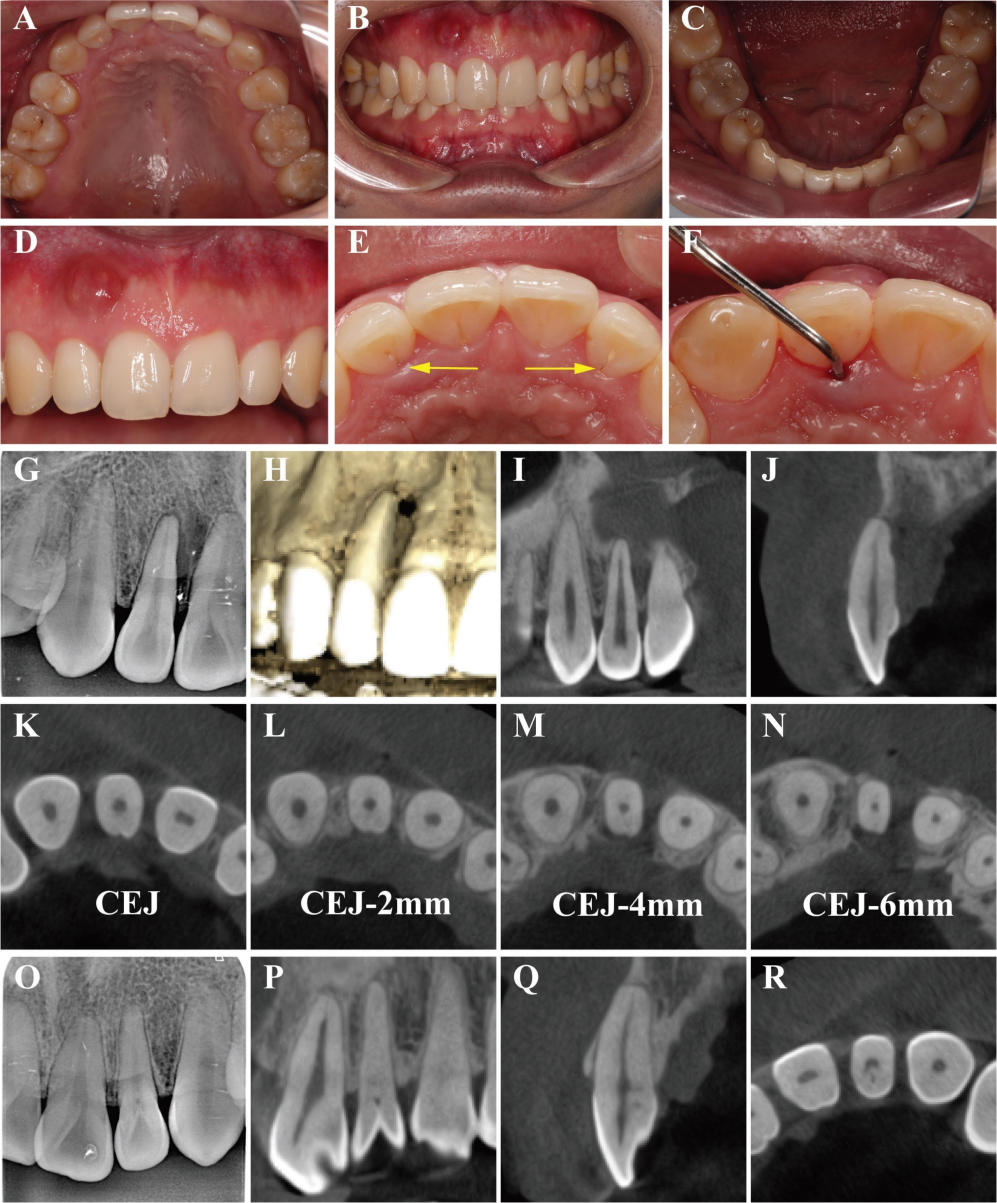
Preoperative diagnostic records: Intraoral photographs and radiographic examination. (A) Maxillary view, (B) frontal occlusal view, (C) mandibular view, (D) buccal view showing a sinus tract between Teeth 11 and 12, (E) palatal view revealing the PGG on Tooth 11 and dens invaginatus on Tooth 22 (yellow arrows indicate), (F) the PGG with a probing depth of 10 mm, (G) periapical radiograph demonstrating periapical and mesial radiolucency associated with Tooth 11, (H) 3D reconstruction showing a through‐and‐through bone defect involving the buccal and palatal cortical plates, (I) coronal view revealing a periapical radiolucent area, (J) sagittal view demonstrating palatal bone wall defect, (K–N) axial view (K) lingual concavity at the CEJ, (L) lingual bone wall destruction at 2 mm below the CEJ, (M) buccolingual bone wall loss at 4 mm below the CEJ, (N) concavity extending to 6 mm below the CEJ with buccolingual bone wall loss, (O) periapical radiograph of dens invaginatus, (P–R) CBCT view confirming dens invaginatus in Tooth 22, (P) coronal view revealing an inward concavity on the crown, (Q) coronal view demonstrating concavity ending at the crown, and (R) axial view showing no communication between the concavity and the pulp.

Extraoral examination was unremarkable, Tooth 12 exhibited moderate percussion tenderness, normal mobility, and a suppurating buccal sinus tract, with no caries or fractures. Periodontally, generalized probing was physiological except for a distinct 10‐mm palatal pocket on 12 associated with a palpable groove. Crucially, pulp sensibility (cold and electric) remained normal for teeth 12, 11, and 22 (Figure 1A–F).

To fully evaluate the 3D extent of the anatomical trajectory of the groove, CBCT was acquired (Planmeca ProMax 3D Mid; 10 × 10 cm FOV, 0.2 mm voxel, 90 kV, 8 mA), revealing a developmental groove on Tooth 12 extending 6 mm apical to the CEJ. Tapering apically, it did not encroach upon the root canal space. Extensive mesio‐bucco‐palatal bone loss with discontinuous cortical plates surrounded the defect (Figure 1G–N). Incidentally, Tooth 22 exhibited an Oehlers Type I dens invaginatus without periapical pathosis (Figure 1O–R).

Primary endodontic pathosis, vertical root fracture, and primary periodontitis were excluded based on normal pulp vitality, intact CBCT findings, and strictly localized periodontal breakdown, respectively. The definitive diagnosis for Tooth 12 was a periodontal abscess secondary to a PGG, and for Tooth 22 was dens invaginatus (Oehlers Type I). The buccal sinus tract was secondary to the palatal defect, draining through the extensive through‐and‐through cortical bone loss. With written consent obtained, the treatment plan featured initial subgingival debridement followed by microsurgery to seal the groove, stimulate periodontal regeneration, and preserve the vital pulp.

**Figure 2:**
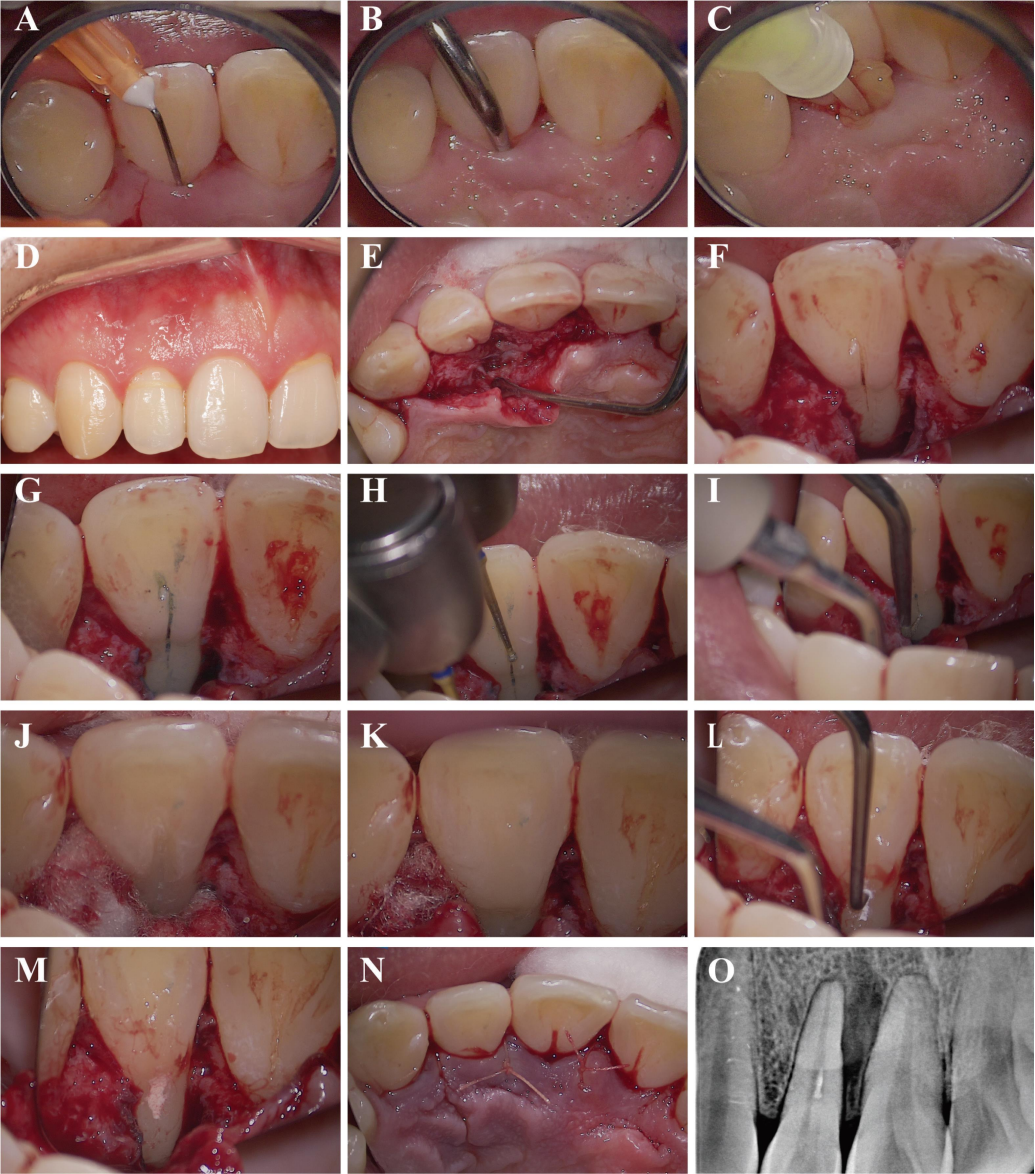
(A) The presurgical management and the microsurgical intervention. (A) Irrigation with sterile saline, (B) ultrasonic subgingival scaling, (C) placement of periocline ointment into periodontal pockets, (D) the buccal sinus tract relieved at 1 month, (E) full‐thickness flap reflection, (F) selective curettage of granulation tissue, (G) methylene blue staining and exploration of the radicular groove, (H) coronal groove preparation (relative to CEJ) using a round bur, (I) apical groove preparation (relative to CEJ) using ultrasonic tips, (J) final cavity preparation completion, (K) coronal groove restoration with composite resin, (L) apical groove obturation with iRoot BP Plus, (M) definitive groove sealing, (N) suturing with absorbable material, and (O) immediate postoperative periapical radiograph.

All procedures were performed under 8–16x microscopic magnification (Zumax Medical, Suzhou, China). Following local infiltration anesthesia with 4% articaine with 1:100,000 epinephrine (Primacaine; Pierre Rolland, Merignac, France), ultrasonic subgingival scaling was performed, and minocycline HCl 2% ointment (Periocline; Sunstar, Osaka, Japan) was applied to the periodontal pocket (Figure 2A–C). At the 1‐month follow‐up, the buccal sinus tract had relieved; however, the deep palatal pocket persisted. Microsurgical treatment was subsequently initiated. A full‐thickness mucoperiosteal flap was raised via a palatal incision extending from Tooth 11 to 13. Under microscopic magnification, the boundaries of the selective curettage were meticulously established utilizing a CBCT‐guided and micromeasured approach. Preoperative CBCT sagittal slices were utilized to precisely measure the distance from the deepest extent of the bony defect to the radiographic apex. Intraoperatively, a periodontal probe was used under the microscope to continuously cross‐reference this depth during the curettage. The inflamed granulation tissue was enucleated until the predefined anatomical safe zone—approximately 1–2 mm short of the radiographic apex—was reached, thereby avoiding mechanical trauma to the vital apical neurovascular bundle. Subsequently, Methylene blue dye (Jumpcan, China) was applied and rinsed with saline to delineate the complex anatomical trajectory of the radicular groove. Then the PGG was carefully prepared using a high‐speed round bur for the coronal portion, and ultrasonic tips for the radicular portion. To practically execute the two‐segment restoration and manage the conflicting moisture and bonding prerequisites of the materials, meticulous regional isolation and hemorrhage control were performed. Complete hemostasis of the adjacent bony defect was achieved using cotton pellets adsorbed with epinephrine (CGE Healthcare, China). The coronal segment (cervical to the CEJ) was dried with a gentle air stream to satisfy the strict hydrophobic conditions required for resin adhesion. A self‐etching adhesive (3M, United States) was applied, and the coronal defect was restored with composite resin (F00, Shofu, Japan). After irrigation, excess moisture in the radicular segment (apical to the CEJ) was blotted with sterile cotton pellets. Then, the defect was filled with iRoot BP Plus (Innovative BioCeramix Inc, Canada). Finally, the flap was repositioned and sutured using absorbable sutures (Ethicon, United States) (Figure 2D–O).

**Figure 3:**
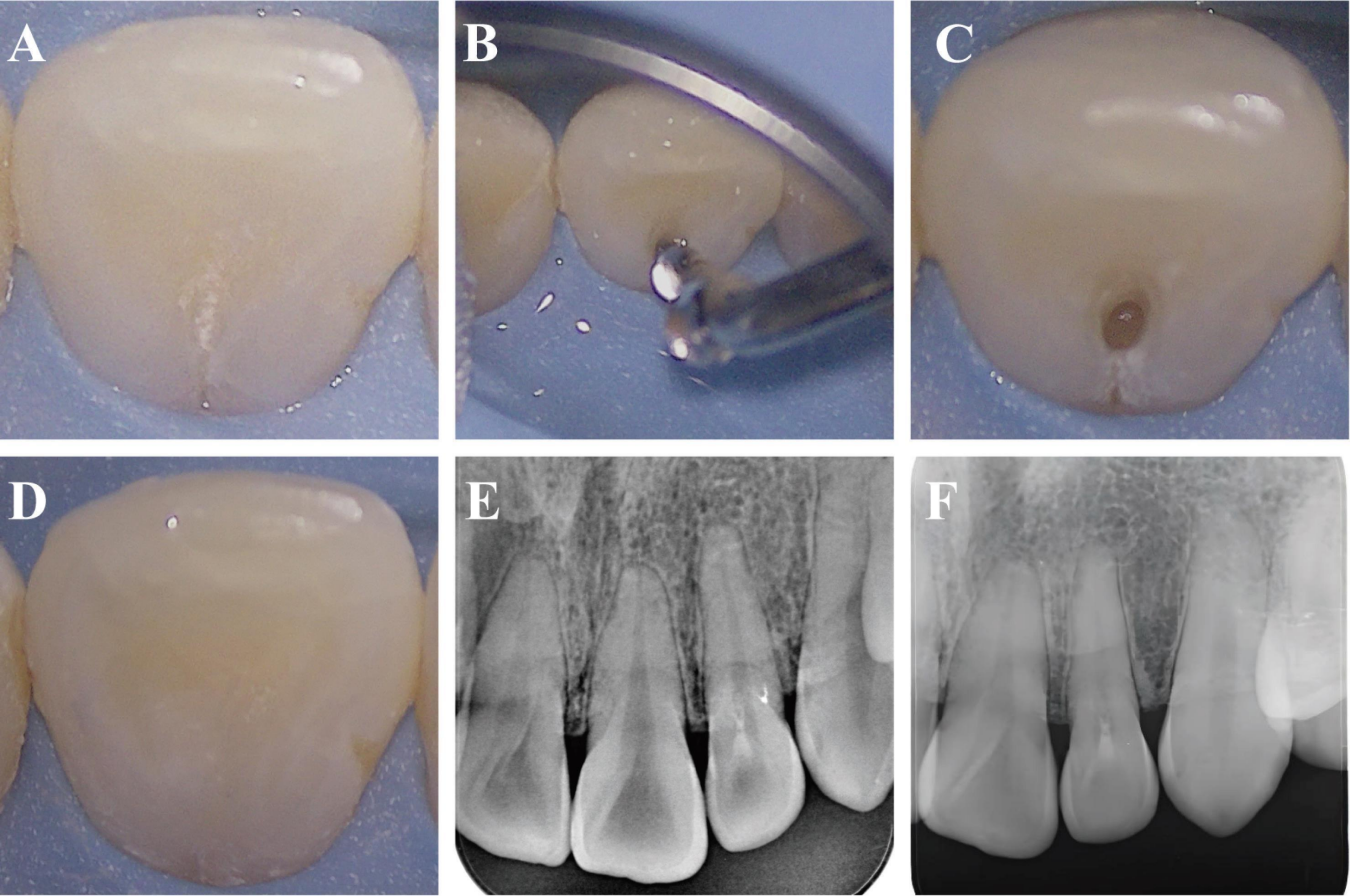
Dens invaginatus management. (A) Rubber dam isolation, (B) ultrasonic debridement of the invagination tract, (C) confirmation of complete debridement under magnification, (D) definitive sealing with composite resin, (E) immediate postoperative periapical radiograph, (F) 2‐year postoperative radiograph.

One week postoperatively, the gingiva exhibited excellent healing, and the sutures were removed. At the same appointment, under rubber dam isolation, the dens invaginatus (Oehlers Type I) on Tooth 22 was addressed; the invagination tract was debrided with ultrasonic tips and then definitively restored with composite resin (F00, Shofu, Japan) to seal the defect (Figure 3).

**Figure 4:**
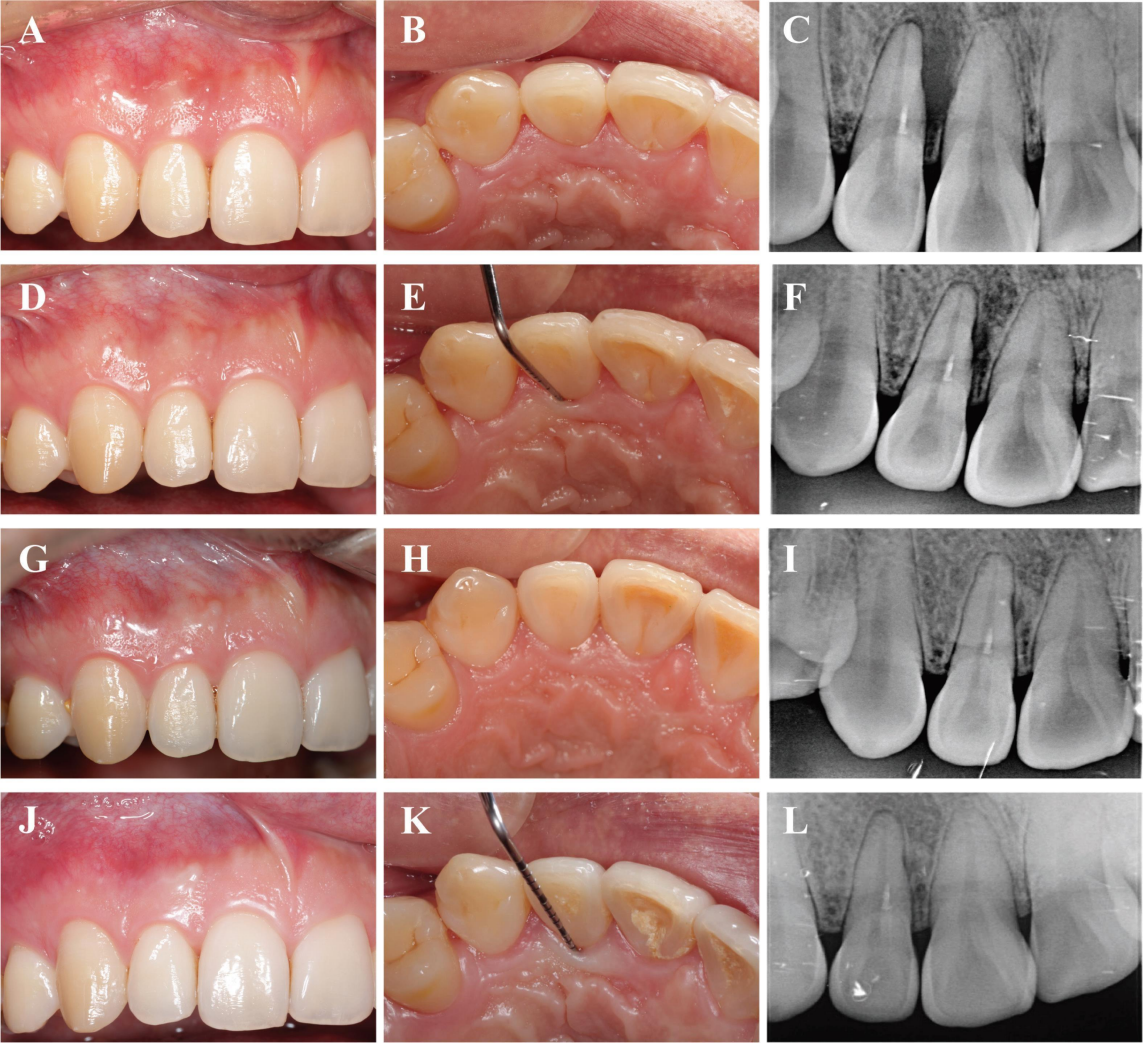
Follow‐up imaging. (A–C) 1‐month postoperative examination. (A) Buccal view showing healing of the sinus tract; (B) palatal view unremarkable; (C) radiograph showing the filling material in place, with a mesial radiolucency; (D–F) 4‐month postoperative examination. (D) Buccal view; (E) palatal view showing a probing depth of 3 mm; (F) radiograph showing decreased extent of the mesial radiolucency; (G–I) 1‐year postoperative examination; (G) buccal view; (H) palatal view; (I) radiograph showing increased mesial radiolucency; (J–L) 2‐year postoperative examination. (J) Buccal view; (K) palatal view showing a probing depth of 2 mm; (L) radiographs showing complete radiolucency resolution and excellent material adaptation.

Postoperative healing was uneventful. Throughout the 2‐year follow‐up period (1, 4, 12, and 24 months), Tooth 12 remained asymptomatic with normal pulp vitality. At 2 years, clinical examination revealed physiological probing depths (< 3 mm) without bleeding, whereas radiographs confirmed complete radiolucency resolution and excellent material adaptation. The patient was highly satisfied with the outcome (Figure 4).

## 3. Discussion

Successful PGG management relies on three principles: microbial elimination, durable sealing of the endodontic‐periodontal communication, and facilitation of periodontal and osseous regeneration [11].

The initial assessment focuses on evaluating pulpal status. When the vital pulp remains anatomically distinct from the PGG, the preferred strategy is managing the groove as an isolated periodontal defect through meticulous debridement of local irritants and granulation tissue, strictly preserving pulp vitality [12]. In this case, despite the presence of a through‐and‐through bone defect on the mesial aspect of the apical region, Tooth 12 remained vital. Complete curettage risked severing the apical neurovascular bundle, compromising vitality. Furthermore, although traditionally excised as pathological, granulation tissue is now recognized to possess significant regenerative potential due to resident pluripotent stem cells [13]. Studies have indicated that granulation tissue contains abundant multipotent stem cells expressing embryonic, neurogenic, and endothelial markers, conferring significant regenerative capacity [14]. Consistently, retaining this tissue during periodontal and peri‐implant surgeries yields favorable healing [15, 16]. Therefore, “selective curettage” was performed to eliminate pathological tissue while preserving the apical neurovascular bundle. This microsurgical approach [9, 15], which removes only 50%–70% of granulation tissue, is typically favored over complete enucleation when operating near critical anatomical structures instead of complete lesion enucleation. By effectively eliminating pathology while retaining apical neurovascular and progenitor tissues, this approach achieved complete osseous healing. To our knowledge, this represents the first successful use of selective curettage to manage a severe PGG without compromising the pulp, introducing a novel therapeutic concept. However, the application of this conservative microsurgical approach may have strict anatomical and pathological limitations, depending on the localization and extension of the PGG. First, the apical extension of the groove must be limited; grooves extending too close to the root apex present severe visual restrictions and extreme operational difficulty for proper debridement and sealing. Second, the inward invagination depth of the PGG must not be in close proximity to the root canal system; dentin removal during groove preparation may lead to irreversible pulpal injury or exposure.

Because cervical PGGs act as conduits for bacterial invasion, they frequently precipitate localized periodontitis [14] or combined endodontic‐periodontal lesions when intersecting with accessory canals [15, 16]. Consequently, achieving a hermetic seal with an appropriate material is paramount for a favorable prognosis. Historically, materials including amalgam, glass ionomer cement, composite resin, and mineral trioxide aggregate (MTA) have been utilized.

The two‐segment restoration method [5] employed in this case—utilizing composite resin coronally and a bioceramic material (iRoot BP Plus) apically to the CEJ—offers significant biomechanical and biological advantages for managing complex anatomical defects. This technique addresses the distinct functional and microbial environments of the coronal and apical portions of the defect. The resin provides a wear‐resistant seal against biofilms and occlusal forces [16, 17]. Apically, the biocompatible and hydraulic bioceramic ensures a hermetic seal against irregular dentin, stimulating cementum‐like apposition and periodontal regeneration [18–21]. The resin material provides excellent sealing, retention, aesthetics, and wear resistance. Meanwhile, the bioceramic material offers an effective sealing barrier, protects the pulp, and promotes periodontal tissue regeneration [21]. Unlike single‐material approaches that compromise either coronal seal longevity or apical biointegration, this region‐specific two‐segment restoration maximizes long‐term prognosis and structural integrity in minimally invasive endodontic‐periodontal microsurgery.

In endodontic surgery, GTR is often considered for through‐and‐through cortical plate defects to prevent gingival connective tissue from prematurely contacting the root surface [22–24]. But no barrier membrane was utilized here because selective curettage inherently relies on residual granulation tissue. At the 2‐year follow‐up radiographs confirmed radiolucency resolution and lamina dura formation. However, lower crestal bone density suggests healing via a long junctional epithelium rather than complete periodontal regeneration, warranting extended follow‐up.

Interestingly, the patient presented concurrently with a PGG and dens invaginatus. Both are well‐documented developmental anomalies with a known predilection for maxillary lateral incisors [25]. From an embryological perspective, they occur at different developmental stages. Dens invaginatus originates from an invagination of the coronal enamel organ into the dental papilla prior to calcification [26, 27], whereas PGG represents a localized infolding of Hertwig’s epithelial root sheath during root morphogenesis [1, 28]. Despite their distinct embryological origins, their concurrent presence in this patient suggests a potential common genetic or morphogenetic susceptibility that warrants further molecular investigation.

## 4. Conclusion

This report demonstrates the successful management of a severe PGG extending to the root apex, despite an initially poor prognosis. At the 2‐year follow‐up, the patient remained asymptomatic with significant radiographic resolution of the lesion. Ultimately, combining selective curettage to preserve vital apical tissues with a two‐segment restoration provides a predictable, biologically driven approach for complex palatogingival grooves.

## Author Contributions

**Shuting Zeng:** conceptualization, data curation (acquisition of clinical data, patient history, and follow‐up documentation) and writing—original draft (drafting the core clinical narrative and treatment protocol). **Simin Guo:** methodology (comprehensive literature search and synthesis regarding concurrent developmental anomalies) and writing—review and editing (critical intellectual revision and restructuring of the discussion). **Zilong Deng:** conceptualization, investigation (performed the core complex microsurgical interventions and clinical operations), supervision (provided critical clinical guidance), and writing—original draft (drafting the radiographic and morphological sections). **Rongyang Ma:** validation (independent verification of the diagnostic rationale, 2‐year follow‐up radiographic outcomes, and critical review of the clinical data integrity), writing—review and editing (critical review of the manuscript). **Wanghong Zhao:** supervision (overall clinical oversight and project administration), writing—review and editing (final critical review of the manuscript). **Shuting Zeng**, **Simin Guo**, and **Zilong Deng** contributed equally to this work.

## Funding

This study was supported by the Clinical Research Fund of Nanfang Hospital, Southern Medical University (2021CR011, 2020CR029), the National Natural Science Foundation of China (82100997, 82270965), the Science and Technology Program of Maoming (2022S020), the Science and Technology Program of Guangzhou (202201010983, 2023B03J1253), and Basic and Applied Basic Research Foundation of Guangdong Province (10.13039/501100021171) (2024A1515013037).

## Disclosure

The authors take full responsibility for the clinical accuracy and final content of the publication. All authors gave final approval and agree to be accountable for all aspects of the work. All authors have read and approved the final version of the manuscript. Wanghong Zhao had full access to all of the data in this study and takes complete responsibility for the integrity of the data and the accuracy of the data analysis.

## Ethics Statement

This retrospective case review did not require formal ethics approval in accordance with local guidelines and the journal′s policy. All the treatment stages were in accordance with the Declaration of Helsinki (1965) and its later amendments. This report was written according to the CARE guidelines.

## Consent

Written informed consent was obtained from the patient for publication of this case report and any accompanying images. All personal identifiers have been removed to protect the patient′s privacy.

## Conflicts of Interest

The authors declare no conflicts of interest.

## Supporting information


**Supporting Information** Additional supporting information can be found online in the Supporting Information section. 

## Data Availability

The data that support the findings of this study are available from the corresponding author upon reasonable request.
