# Supplementary material for: Selective Curettage and Two‐Segment Restoration in the Management of a Palatogingival Groove With Vital Pulp—A 2‐Year Follow‐Up Case Report
Source: Case Rep Dent. 2026 Aug 27;2026:9391532. doi: 10.1155/crid/9391532 (PMC13522714; doi:10.1155/crid/9391532)
Supplement: Supplementary file 1 [file CRID-2026-9391532-s001.pdf]

CARE Checklist of information to include when writing a case report

CARE case report guidelines

(cc) BY-NC-ND

Topic	Item	Checklist item description	Reported on Line
Title	1	The diagnosis or intervention of primary focus followed by the words "case report"	Lines 3
Key Words	2	2 to 5 key words that identify diagnoses or interventions in this case report, including "case report"	Lines 18
Abstract (no references)	3a	Introduction: What is unique about this case and what does it add to the scientific literature?	Lines 5-6
	3b	Main symptoms and/or important clinical findings	Lines 6-8
	3c	The main diagnoses, therapeutic interventions, and outcomes	Lines 9-14
	3d	Conclusion—What is the main "take-away" lesson(s) from this case?	Lines 15-17
Introduction	4	One or two paragraphs summarizing why this case is unique (may include references)	Lines 22-41
Patient Information	5a	De-identified patient specific information	Lines 44
	5b	Primary concerns and symptoms of the patient	Lines 45-46
	5c	Medical, family, and psycho-social history including relevant genetic information	Lines 46-48
	5d	Relevant past interventions with outcomes	Not applicable
Clinical Findings	6	Describe significant physical examination (PE) and important clinical findings	Lines 49-53
Timeline	7	Historical and current information from this episode of care organized as a timeline	Not applicable
Diagnostic Assessment	8a	Diagnostic testing (such as PE, laboratory testing, imaging, surveys)	Lines 54-60
	8b	Diagnostic challenges (such as access to testing, financial, or cultural)	Lines 61
	8c	Diagnosis (including other diagnoses considered)	Lines 61-65
	8d	Prognosis (such as staging in oncology) where applicable	Lines 108-113
Therapeutic Intervention	9a	Types of therapeutic intervention (such as pharmacologic, surgical, preventive, self-care)	Lines 66-69
	9b	Administration of therapeutic intervention (such as dosage, strength, duration)	Lines 70-100
	9c	Changes in therapeutic intervention (with rationale)	Not applicable
Follow-up and Outcomes	10a	Clinician and patient-assessed outcomes (if available)	Lines 112
	10b	Important follow-up diagnostic and other test results	Lines 108-112
	10c	Intervention adherence and tolerability (How was this assessed?)	Lines 109
	10d	Adverse and unanticipated events	Not applicable
Discussion	11a	A scientific discussion of the strengths AND limitations associated with this case report	Lines 118-146
	11b	Discussion of the relevant medical literature with references	Lines 147-167
	11c	The scientific rationale for any conclusions (including assessment of possible causes)	Lines 168-175
	11d	The primary "take-away" lessons of this case report (without references) in a one paragraph conclusion	Lines 187-192
Patient Perspective	12	The patient should share their perspective in one to two paragraphs on the treatment(s) they received	Lines 112
Informed Consent	13	Did the patient give informed consent? Please provide if requested	Yes √ No
